# Proteomic Characterization of *Helicobacter pylori* CagA Antigen Recognized by Child Serum Antibodies and Its Epitope Mapping by Peptide Array

**DOI:** 10.1371/journal.pone.0104611

**Published:** 2014-08-20

**Authors:** Junko Akada, Masumi Okuda, Narumi Hiramoto, Takao Kitagawa, Xiulian Zhang, Shuichi Kamei, Akane Ito, Mikiko Nakamura, Tomohisa Uchida, Tomoko Hiwatani, Yoshihiro Fukuda, Teruko Nakazawa, Yasuhiro Kuramitsu, Kazuyuki Nakamura

**Affiliations:** 1 Department of Biochemistry and Functional Proteomics, Yamaguchi University Graduate School of Medicine, Ube, Yamaguchi, Japan; 2 Sasayama Medical Center, Hyogo College of Medicine, Sasayama, Hyogo, Japan; 3 Department of General Medicine and Community Health Science, Hyogo College of Medicine, Nishinomiya, Hyogo, Japan; 4 Technical Research Laboratory, Toyo Kohan Company, Ltd., Kudamatsu, Yamaguchi, Japan; 5 Innovation Center with University-Industry-Public Cooperation, Organization for Research Initiatives, Yamaguchi University, Tokiwadai, Ube, Yamaguchi, Japan; 6 Department of Molecular Pathology, Oita University, Faculty of Medicine, Oita University, Yufu, Oita, Japan; 7 Wakayama-Rosai Hospital, Wakayama, Wakayama, Japan; 8 Department of Microbiology and Immunology, Yamaguchi University Graduate School of Medicine, Ube, Yamaguchi, Japan; Veterans Affairs Medical Center (111D), United States of America

## Abstract

Serum antibodies against pathogenic bacteria play immunologically protective roles, and can be utilized as diagnostic markers of infection. This study focused on Japanese child serum antibodies against *Helicobacter pylori*, a chronically-infected gastric bacterium which causes gastric cancer in adults. Serological diagnosis for *H. pylori* infection is well established for adults, but it needs to be improved for children. Serum samples from 24 children, 22 *H. pylori* (*Hp*)-positive and 2 *Hp*-negative children, were used to catalogue antigenic proteins of a Japanese strain CPY2052 by two-dimensional electrophoresis followed by immunoblot and LC-MS/MS analysis. In total, 24 proteins were identified as candidate antigen proteins. Among these, the major virulence factor, cytotoxin-associated gene A protein (CagA) was the most reactive antigen recognized by all the *Hp*-positive sera even from children under the age of 3 years. The major antigenic part of CagA was identified in the middle region, and two peptides containing CagA epitopes were identified using a newly developed peptide/protein-combined array chip method, modified from our previous protein chip method. Each of the epitopes was found to contain amino acid residue(s) unique to East Asian CagA. Epitope analysis of CagA indicated importance of the regional CagA antigens for serodiagnosis of *H. pylori* infection in children.

## Introduction

Approximately half of the world’s population is estimated to harbor *Helicobacter pylori*, a pathogenic gastric bacterium. The infection is primarily established during childhood and persists for decades unless eradicated. *H. pylori* infection is the major cause of chronic gastritis, and linked to severe gastric diseases such as peptic ulcers, MALT lymphoma, and gastric adenocarcinoma [1]. *H. pylori* translocates a virulence factor, cytotoxin-associated gene A protein (CagA), into gastric epithelial cells by the type IV secretion system, resulting in activation of cell signaling pathways which trigger oncogenic cellular phenotypes and immune responses in host cells [2]. CagA has been originally described as an immunodominant antigen recognized by serum antibodies of gastric disease patients [3], [4]. It was recently revealed in mouse models that CagA specific-T cells induce preneoplastic immunopathology, whereas CagA-dependent T-cell priming induces regulatory T-cell differentiation [5], [6]. Meta-analyses of case-control studies revealed that CagA seropositivity is associated with an increased risk of developing gastric cancer [7], [8]; however, the role of CagA in host adaptive immunity is still not fully understood.

Early diagnosis of *H. pylori* infection is important for the control of gastric diseases in East Asian countries where the incidence of gastric cancer is high [9]. Pre-screening of high-risk individuals for gastric cancer performed during health check-ups and in a cohort study in Japan indicated that a combination of the serum anti-*H. pylori* IgG titer and the ratio of pepsinogen I to II levels is promising for pre-screening [10], [11].

Serological diagnosis of *H. pylori* infection is necessary for children because invasive endoscopy is not suitable for this population. In most cases, infected children are asymptomatic, but some patients develop gastritis and peptic ulcer diseases [12], [13]. Moreover, some non-gastric diseases, such as iron-deficiency anemia and epilepsy, have been suggested to be associated with *H. pylori* infection [14]–[16]. Serological diagnosis of *H. pylori* for children, however, has been problematic for Japanese children; we found that the diagnostic performance of an enzyme immune assay (EIA) kit based on Japanese strain-derived high-molecular-weight cell-associated protein (JHM-CAP kit) was much better than that based on U.S. strain-derived high-molecular-weight cell-associated protein (HM-CAP kit) [17], although the performances of the two kits for adult populations were not much different [18]. Such a clear difference between the two kits for diagnosis of *H. pylori* infection in Japanese children was shown to be attributed to the 100-kDa antigen protein contained only in JHM-CAP, which was later identified as a fragmented CagA protein [19]. Intriguingly, 100-kDa CagA fragments were also identified in gastric cells and phagocytic cells after infection by western *H. pylori* strain [20], [21].

Comprehensive immunoproteomic analyses have been performed on sera from adult patients with gastric and non-gastric diseases [22]–[31]. However, sera from *H. pylori-*infected children have not been studied yet. In this study, we performed two-dimensional antigenic protein analysis to catalogue *H. pylori* antigen proteins that reacted with IgG antibodies in sera from *H. pylori* (*Hp*)*-*positive Japanese children. And then, we focused on the highly immunodominant protein CagA, since it reacted with all the serum samples tested. We identified two epitopes in the middle region of Japanese CagA which have unique sequences for East Asian CagA, using a newly developed peptide/protein-combined array method. This epitope mapping method was modified from the previously described method of protein array on maleimide-incorporated diamond-like carbon (DLC)-coated silicon chip [32], [33]. Our findings indicated that East Asian-specific CagA antigens may be useful for detecting *H. pylori* infection in children of East Asian countries where gastric cancer is prevailing.

## Results

### Detection of antigenic *H. Pylori* protein spots on two-dimensional (2-D) immunoblots probed with IgG in sera from children

**Figure 1:**
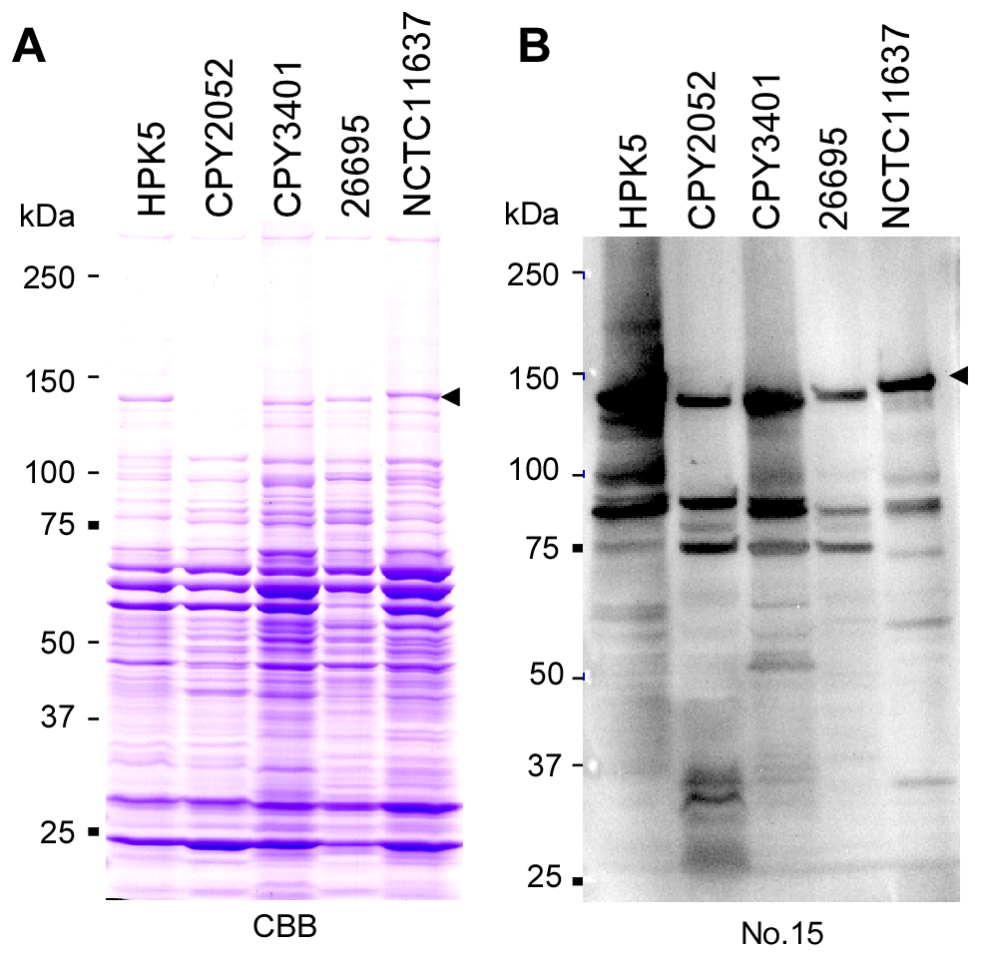
Immunoblot analysis of whole-cell lysate of East Asian and western *Helicobacter pylori* strains using serum from Japanese child. **A**. A Coomarssie Brilliant Blue R-250 (CBB)-stained SDS-PAGE gels containing whole-cell lysate of three East Asian *Helicobacter pylori* strains (HPK5, CPY2052, and CPY3401) and two western *H. pylori* strains (26695 and NCTC11637). The 130–140 kDa band (black arrowhead) is CagA by LC-MS/MS. **B**. Representative immunoblot. A SDS-PAGE gel, was blotted onto PVDF membrane, and probed with diluted serum No. 15 from a child (4-year-old) with juvenile rheumatoid arthritis. The serum reacted with CagA (black arrowhead) and several other proteins with low molecular weights.

To select a strain of *H. pylori* suitable for serological proteome analysis, cell lysate samples of three Japanese strains, HPK5, CPY2052, and CPY3401, as well as two western strains, 26695 and NTCT11637, were subjected to SDS-PAGE analysis followed by immunoblotting with serum No. 15 from a 4-year-old Japanese child with juvenile rheumatoid arthritis (Figure 1, Table S1 in Supporting Information). Japanese strains showed a distinct 130-kDa band of CagA which is highly immunoreactive as reported previously [17]. Among these, CPY2052, a low CagA producer was selected for 2-D immunoblot analysis, because large amounts of CagA of other strains might disturb the immunoblot analyses.

**Figure 2:**
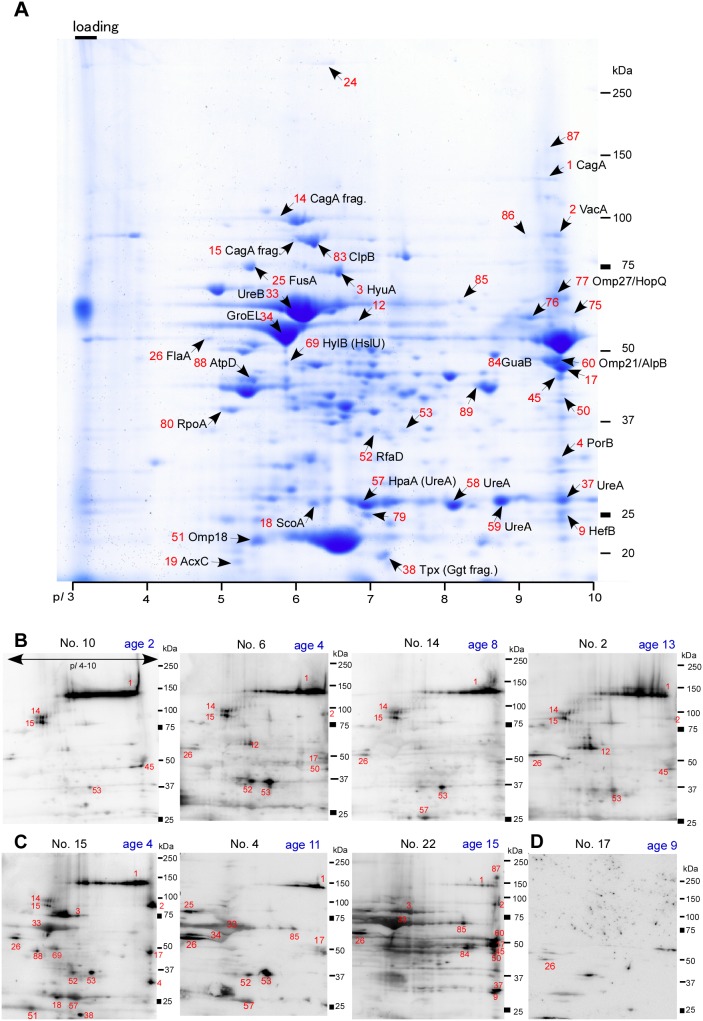
A CBB stained 2DE pattern of whole-cell lysates of *H. pylori* strain CPY2052 and representative images of 2D-immunoblots probed with sera from children. **A.** See Pico CBB-stained 2-DE gel containing proteins from *H. pylori* CPY2052. Spots of antigenic proteins recognized by at least one serum sample are indicated by arrows with numbers in red. Abbreviated names of proteins identified by MS analysis (Table 1) are indicated in black. The red number of each spot corresponds to a W number in Table 1. The p*I* and molecular size markers are indicated at the bottom and to the right, respectively, and cup-loading site in IEF is indicated as a black bar at the top, left end of the gel. **B**. Representative images of immunoblots probed with sera from asymptomatic children. **C**. Representative images of immunoblots probed with sera from symptomatic children. Serum No. 15, juvenile rheumatoid arthritis, No. 4, epilepsia, and No. 22, iron-deficiency anemia, (Table S1). D. A image of immunoblot probed with serum from *Hp*-negative child. Serum No. and age of children are indicated at the top of each panel.

A 2-D gel exhibiting stained proteins from CPY2052 and representative immunoblots are shown in Figure 2. Forty immunoreactive protein spots detected with 22 *Hp*-positive Japanese children were further identified by LC MS/MS (Figure 2A, identified by numbered arrows with abbreviated names). As shown by representative images, sera from asymptomatic children reacted with a relatively small number of *H. pylori* protein spots (Figure 2B) as compared to those from symptomatic children who had some non-gastric diseases (Figure 2C). Intriguingly, highly expressed proteins in CPY2052 such as urease subunits (UreA and UreB) and a chaperone protein GroEL (also called as HspB) did not react with sera from asymptomatic children (Figure 2A, 2B and Table S1). A serum sample from a non-infected child reacted only with flagellin subunit, FlaA (Figure 2D).

Thirty antigenic proteins of Japanese strain CPY2052 probed by 22 serum samples from *Hp*-positive Japanese children of 2 to 13 years of age were identified by LC-MS/MS with available databases of *H. pylori* (Table 1, spot numbers in Figure 2A were given prefix W). Spot W14 and W15 were both identified as CagA fragments, while spot W37, W58, and W59 were all identified as urease subunit UreA. Spot W38, W57, and W69 contained two proteins. FlaA was excluded from the candidate antigen protein list because of its non-specific reaction. In summary, 40 protein spots were distinguished by 2D-immunoblot analysis, and proteins of 27 spots were identified by LC-MS/MS to propose 24 candidate antigenic proteins, including 9 newly identified proteins. It is noteworthy that CagA, CagA fragments, FlaA, spot W53 (not identified), Omp21/AlpB and VacA were recognized by more than 50% of serum samples from *Hp*-positive children (Table 1). Importantly, CagA was recognized by all the serum samples from *Hp*-positive children.

**Table 1:** Candidates of *Helicobacter pylori* antigenic proteins recognized by IgG antibodies in sera of Japanese *H. pylori*-positive children as determined by LC-MS/MS.

	2DE-gel				MS results						Genome	
Spot No.	MW(kDa)	p*I*	Protein Name^1)^	Distinctivepeptide (#)^2)^	MS/MSSearchScore^3)^	% AACoverage	ProteinMW(Da)	Proteinp*I*	Database^4)^	IB^5)^	HP No.in 26695	No. of Hp (+)sera reacted^6)^n = 22
W1	130	9.5	Cytotoxin associate gene A (CagA)	6	74.9	7	130708	8.3	NCBI	CagA	HP0547	22 (100%)
W2	85	10.0	Vacuolating cytotoxin A (VacA)	1	11.2	1	93255	9.2	NCBI	VacA	HP0887	11 (50%)
W3	75	6.5	Hydantoin utilization protein A (HyuA)	21	316.3	31	78533	6.5	NCBI		HP0695	2
W4	30	9.5	Ferredoxin oxidoreductase subunitbeta (PorB)*	2	23.1	7	34987	8.5	NCBI		HP1111	5
W9	20	9.5	Efflux transporter (HefB)*	2	22.0	4	26748	8.7	NCBI		HP0606	7
W14	100	6.0	CagA fragment	3	27.2	4	130829	8.1	SP & EA	CagA	HP0547	18 (82%)
W15	85	6.0	CagA fragment	0	Immunodetection only					CagA	HP0547	
W18	26	6.0	Succinyl-CoA:3-ketoacid-coenzyme Atransferase subunit A (ScoA/YxjD)	4	58.4	23	25325	5.9	NCBI		HP0691	2
W19	19	5.2	Acetone carboxylasegamma subunit (AcxC)*	2	30.0	14	19671	5.1	NCBI		HP0697	1
W25	75	5.0	Elongation factor G (FusA)*	17	231.6	27	77022	5.2	SP		HP1195	4
W26	51	4.8	Flagellin (FlaA)	5	62.1	7	53342	5.8	NCBI		HP0601	16 (73%)**
W33	62	6.5	Urease subunit beta (UreB)	19	296.0	36	61684	5.6	SP		HP0072	5
W34	55	5.8	60 kDa chaperonin (GroEL)	7	117.3	17	58264	5.6	SP		HP0010	4
W37	26	9.5	Urease subunit alpha (UreA)	16	206.4	46	26540	8.5	SP		HP0073	5
W38	19	7.7	Probable thiol peroxidase (Tpx)*	4	53.0	19	18292	7.7	SP		Hp0390	3
			Gamma-glutamyltranspeptidase(Ggt) fragment*	2	22.6	2	61090	9.2	NCBI		HP1118	
W51	21	5.3	Outer membrane protein (Omp18)	5	52.5	20	20042	5.9	NCBI		HP1125	3
W52	35	7.2	ADP-L-glycero-D-manno-heptose-6-epimerase (RfaD)*	2	31.4	7	37696	6.1	NCBI		HP0859	4
W57	26	6.9	Urease subunit alpha (UreA)	11	147.7	54	26568	8.5	NCBI		HP0073	7
			Flagellar shearth adhesin (HpaA)	8	100.2	37	29130	8.6	NCBI		HP0410	
W58	25	8.0	Urease subunit alpha (UreA)	3	42.4	13	26540	8.5	SP		HP0073	same as W37
W59	26	8.8	Urease subunit alpha (UreA)	11	160.4	40	26568	8.5	NCBI		HP0073	
W60	50	9.5	Outer membrane protein adhesin(Omp21/AlpB)	2	28.3	4	56638	9.2	NCBI		HP0913	12 (55%)
W69	49	5.7	Methyl-accepting chemotaxis protein(MCP/HylB)	4	61.0	11	48384	5.9	NCBI		HP0599	2
			Heat shock protein (HslU)*	4	48.3	8	50191	5.5	NCBI		HP0516	
W77	70	10.0	Outer membrane protein(Omp27/HopQ)*	6	85.0	11	69803	9.3	NCBI		HP1177	3
W80	40	5.0	DNA-directed RNA polymerasesubunit alpha (RpoA)	16	209.7	43	38499	5.0	NCBI		HP1293	1
W83	100	6.5	Chaperone protein ClpB (ClpB)	7	118.0	10	96684	6.0	SP		HP0264	1
W84	42	8.5	Inosine 5′-monophosphatedehydrogenase (GuaB)	14	196.4	37	51689	7.2	NCBI		HP0829	2
W88	45	5.3	F0F1 ATP synthase subunit beta (AtpD)	12	191.2	33	51479	5.3	NCBI		HP1132	1

1) Protein names with * are newly identified *H. pylori* antigenic protein candidates in this study. Abbreviated names are indidcated in parentheses.

Alternative name is indicated after/. W38, W57 and W69 spots included two antigen candidate proteins with high MS scores.

2) Proteins which were identified from more than two distinctive peptides by Spectrum Mill MS Proteomics Workbench, except W2 and W15.

3) Proteins which were identified as more than 20 MS/MS Search Score by Spectrum Mill MS Proteomics Workbench, except W2 and W15.

4) SP: Swiss Prot, EA: East-Asia 7 strains, othrers are all by NCBI.

5) Identification by immunoblot analysis using wild type and knockout strains of the coressponding genes.

6) FlaA was also reacted with two of *H. pylori*-negative child serum samples, not included in this number**.

### Identification of epitope region of CagA protein

**Figure 3:**
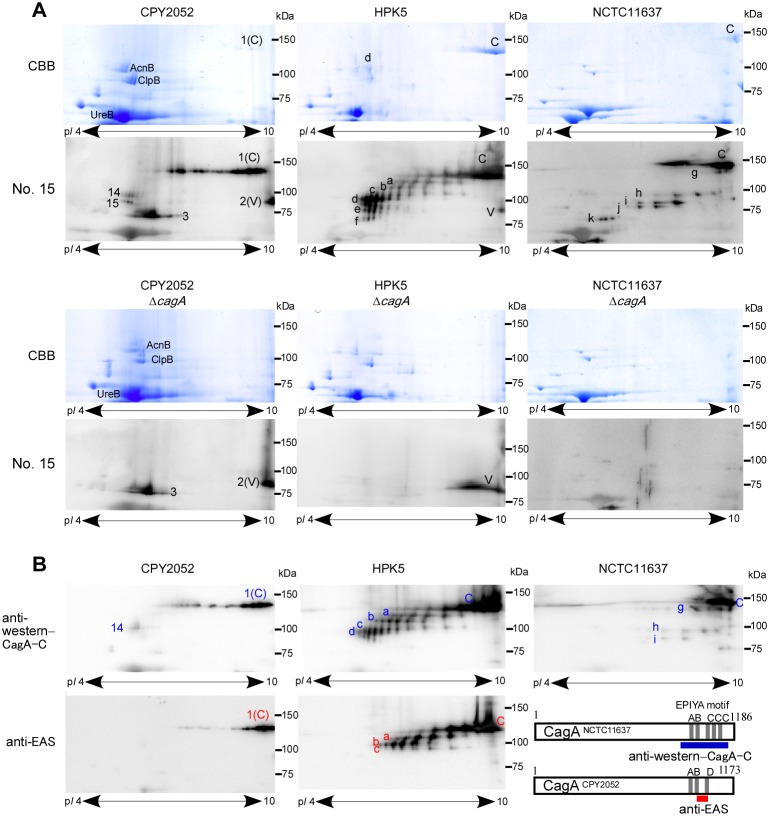
2D-immunoblot analysis of CagA and CagA fragments with serum from *H. pylori-*positive child and CagA-specific antibodies. **A**. See Pico CBB-stained 2-DE gels and immunoblots of CPY2052, HPK5 and NCTC11637 and their *cagA* deletion strains probed with *Hp*-positive serum No. 15 serum. **B**. Immuoblots probed with antibodies for EPIYA motif-containing fragment of western-CagA (anti-western-CagA-C) and East Asian CagA (anti-EAS). Regions of CagA to produce two antibodies are diagrammed schematically. Spot numbers are corresponded to those in Fig. 2. 1(C) or C is CagA, and 2(V) or V is VacA. Spots of a-f or g-k are CagA-fragments in 2-D immunoblot of HPK5 and NCTC11637 lysate, respectively.

The above 2-D immunoblot analyses indicated that CagA antibodies are the dominant anti-*Hp* antibodies in sera from Japanese *Hp-*positive children. To analyze the antigenic region of CagA and CagA fragments, CPY2052 and two CagA-overproducing strains, Japanese strain HPK5 and Western strain NCTC11637, and their *cagA*-deletion mutants were subjected to 2-D immunoblot analyses. When the 2-D blots of the wild-type strains was probed with serum No. 15, highly immunoreactive bands were observed which were completely absent when the *cagA-*deletion mutants were probed (Figure 3A). Interestingly, the 2-D immunoblotting patterns of HPK5 and NCTC11637 were clearly different. CagA of HPK5 (CagA^HPK5^) showed at least six distinct bands ranging from 75 to 120 kDa in addition to the full-length band, whereas CagA of NCTC11637 (CagA^NCTC11637^) showed the major full-length band followed by weak minor bands.

The antigenic 100-kDa fragment of CagA has previously been reported in Japanese strains [19]. In addition, 100-kDa CagA fragments were detected in gastric cells and phagocytic cells of mice by infecting western *H. pylori* strain P1 [20], [21]. These results suggested that CagA is readily degraded *in vitro* and *in vivo* resulting to produce the 100 kDa-fragment. In accord with this, the 2-D gel of HPK5 shows proteins spots of 130-kDa CagA and 100-kDa CagA fragment (Figure 3A, spot C and spot d, respectively). If this is the case for CagA^HPK5,^ and each of the proteins are assumed to be unstable, anti-CagA antibodies might have reacted with degraded proteins of CagA (Figure 3A, spot a, b, c) as well as of the 100-kDa fragment (Figure 3A, spot e, f). Similar interpretation might also be possible on the immunoblots of CPY2052 (spot 1(C) of CagA, and spot 15 derived from spot 14 of 100-kDa fragment) and NTCT11637 (spot g derived from spot C of CagA, and spot i, j and k derived from spot h of 100-kDa fragment).

We then carried out 2-D immunoblot analyses of CagA using polyclonal antibodies raised against a western CagA C-terminal fragment containing the EPIYA motifs (anti-western CagA-C) and an East-Asian CagA fragment containing a short C-terminal region (anti-EAS) (Figure 3B). The CagA protein and its fragments of strains CPY2052, HPK5 and NTCT11637 were recognized well by anti-western CagA-C and anti-EAS antibodies as expected. However interestingly, smaller fragments observed on the immunoblots with serum No. 15, were not recognized by anti-western CagA-C (spot 15 of CPY2052, e and f of HPK5, and spot j and k of NCTC11637 in Figure 3A vs Figure 3B), or by anti-EAS (spot 14 and 15 of CPY2052 and d, e and f of HPK5). Therefore, regions of East-Asian CagA other than the C-terminus may contain additional epitopes recognized by antibodies in Japanese child sera.

**Figure 4:**
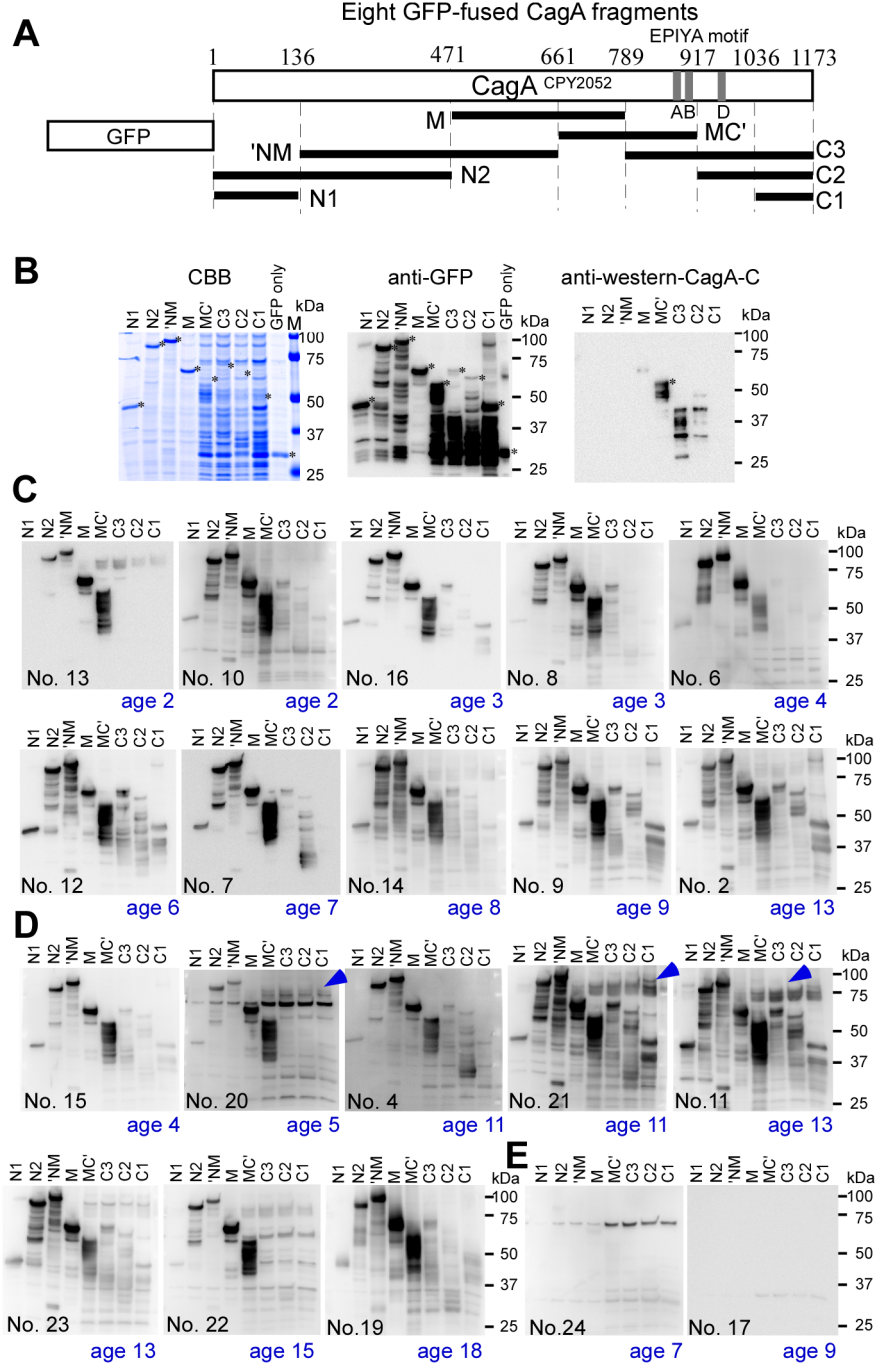
Antigenic region of CagA^CPY2052^ determined by immunoblotting analysis of *E. coli* lysates containing recombinant proteins. **A**. A series of eight distinct CagA fragments fused with GFP at N-terminus expressed in *E. coli*. **B**. CBB-stained SDS-PAGE gel of *E. coli* lysates, immunoblots probed with anti-GFP, and immunoblots probed with anti-CagA C-terminus specific antibody (anti-western-CagA-C). Asterisks in the images indicate each full-length recombinant protein. Four-times more volumes of lysates expressing CagA C-terminal fragments, MC’, C3, C2, and C1, were loaded to compensate the small amount of antigens. **C**. Immunoblots probed with sera from *Hp-*positive, asymptomatic children. **D**. Immunoblots probed with sera from *Hp-*positive symptomatic children. Three highly reactive proteins possibly from *E. coli* are indicated by blue arrowheads. **E**. Immunoblots probed with sera from *Hp-*negative children showing *E. coli*-derived bands similar to those in D.

To localize East Asian strain-specific CagA epitopes, GFP-fused CagA fragments of CPY2052 were expressed in *E. coli* strain BL21 followed by SDS-PAGE and immunoblotting with anti-GFP, anti-western CagA-C antibody, and sera from Japanese children (Figure 4). GFP-fused fragments containing the N-terminal or middle region of CagA (N1, N2, ‘NM, and M) were found to be expressed stably in *E. coli*, whereas the fragments containing the C-terminal region of CagA (MC’, C3, C2 and C1) appeared to be unstable (Figure 4B). When 4 times the volume of lysate of the C-terminal fragments were applied on SDS-PADE, bands at the expected full length appeared together with numerous smaller bands by anti-GFP immunoblotting. Immunoblots of fragment MC’ with anti-western CagA-C antibody showed a full length band followed by several small bands, whereas those of fragment C3 and C2 showed only small bands.

Immunoblot analyses of GFP-fused CagA fragments with sera from *Hp-*positive asymptomatic and symptomatic children with some non-gastric diseases, were carried out (Figure 4C and 4D). Intriguingly, immunoblot patterns probed with sera from children younger than 4-year-old (serum No. 13, No. 10, No. 16, No. 8, No. 6 in Figure 4C and serum No. 15 of Figure 4D) were very similar. Fragment N2, ‘NM, M, or MC’ gave full length and smaller bands, whereas fragment C3, C2, or C1 gave no bands. On the other hand, immunoblot patterns probed with sera from older children tended to have more bands of fragment C3, C2, and C1. Some non-specific bands possibly due to *E. coli* proteins in lysate were observed on immnoblots probed with sera from some symptomatic children (Fig. 4D). Immunoblots with sera from *Hp*-negative children had essentially no band (Figure 4E). The results of immunoblot analysis of CagA fragments with sera from 22 *Hp*-positive children are summarized in Table 2. Based on these results, we concluded that the most frequently-recognized antigenic epitopes of CagA^CPY2052^ at an early stage of infection are present in the middle region covered by fragments ‘NM, M and MC’ (136–917 aa in CagA^CPY2052^, Fig. 4A).

**Table 2:** Antigenic region of CagA^CPY2052^ protein recognized by IgG antibodies in *H. pylori (Hp)*-positive child sera.

GFP-fused CagA fragment in *E. coli* lysate	Number of sera reacted in *Hp*-positive serum samples (%)
N1	9
N2	22 (100%)
NM’	22 (100%)
M	22 (100%)
MC’	22 (100%)
C3	7
C2	9
C1	6
EGFP only	0

### On-chip epitope mapping of antibodies recognizing the CagA middle region

**Figure 5:**
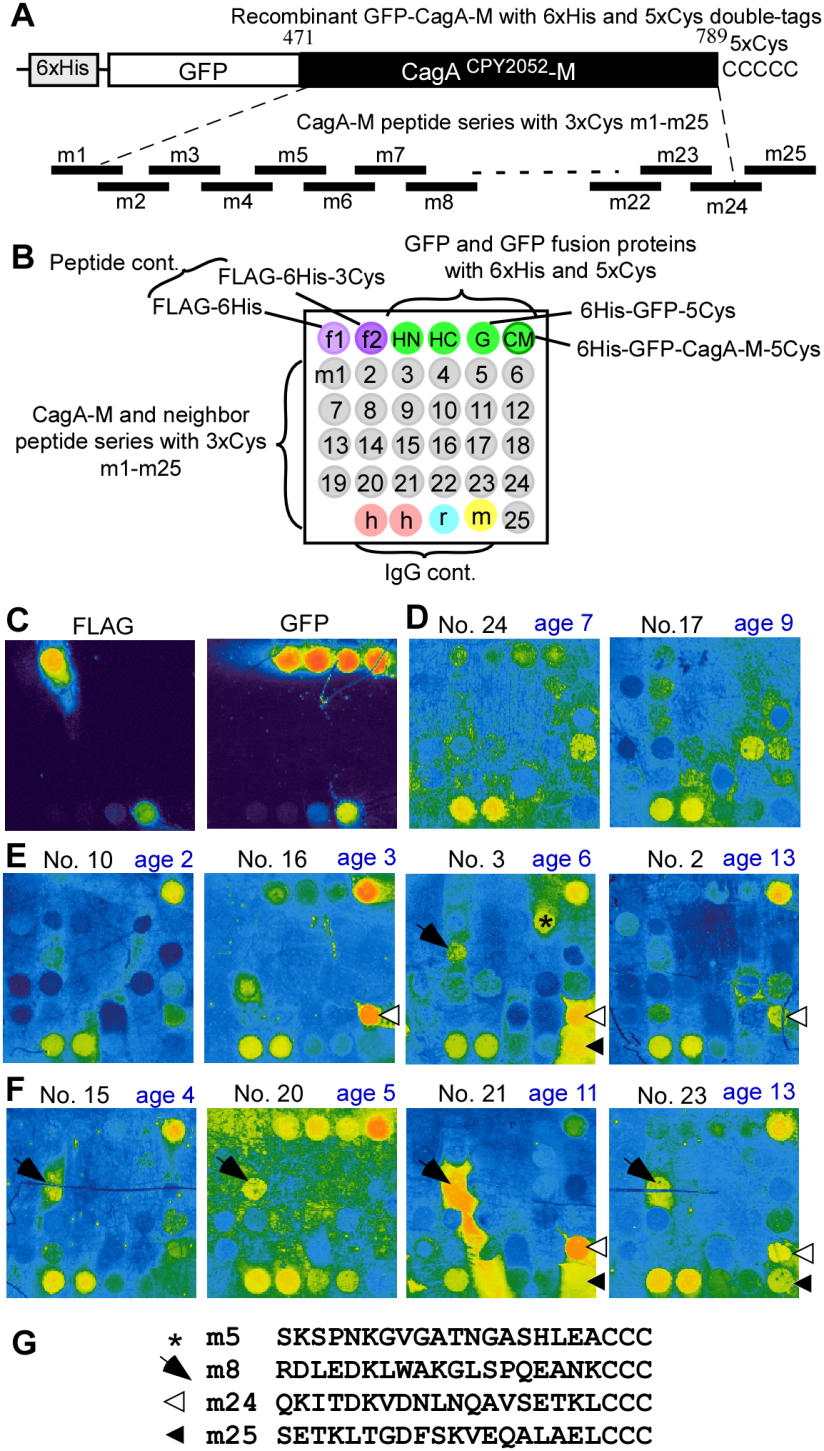
Epitope mapping in the middle region of CagA^CPY2052^ with a peptide/protein array. **A**. A series of CagA^CPY2052^-M peptides contained 19 amino acids tagged with 3xCys at the C-terminus. **B**. Layout of a peptide/protein array chip. Each spot (200 µm in diameters) contained 10 fmol of peptide or 128 fmol of protein. IgG spots each containing 64 fmol of protein were used as positive controls for detection of fluorescent second antibodies (h, anti-human IgG; r, rabbit IgG; m, mouse IgG). Each purified recombinant protein with a 5xCys-tag or peptide with a 3xCys-tag was spotted and immobilized on 3×3 mm maleimide-incorporated DLC chip. **C-F**. Full-color images of antibody detection on peptide/protein chips. **C**. Image of chips probed with anti-FLAG and anti-GFP mouse antibodies. **D**. Images of chips probed with sera from *Hp-*negative children. **E**. Images of chips probed with sera from *Hp-*positive asymptomatic children, **F**. Images of chips probed with sera from *Hp-*positive symptomatic children. **G**. Peptide sequences that reacted with *Hp-*positive sera as indicated on images of E and F with asterisk, arrow, white arrowhead, and black arrowhead.

To identify epitopes in the middle region of CagA^CPY2052^ (CagA-M), a peptide/protein-combined array method was developed. Based on our previous protein array method, six-histidine (6xHis)- and five-cysteine (5xCys)- double tagged recombinant CagA proteins were constructed. The proteins were purified via 6xHis-tag, and immobilized on the maleimide-incorporated DLC chip surface via 5xCys-tag. Then twenty-five peptides (m1 thru m25) tagged with 3xCys were fixed on maleimide-DLC chips together with control peptides and control proteins, including GFP, and GFP-fused Cag-M tagged with 5xCys (Figure 5A and 5B). As shown in Figure 5C, FLAG-6xHis-3Cys, but not FLAG-6xHis, was clearly recognized by anti-FLAG antibody visualized with fluorescent anti-mouse IgG, confirming that 3xCys was necessary to immobilize the peptide on the chip. In addition, all the control proteins were recognized by anti-GFP antibody visualized with fluorescent Alexa-633-conjugated anti-rabbit IgG.

All the sera from *Hp*-positive children reacted with the GFP-fused CagA-M, whereas no signal appeared when sera from *Hp*-negative children were tested (Figure 5D). Serum No. 10 from a 2-year-old child reacted only with GFP-fused CagA-M, while other samples from older children also reacted with peptides such as m5, m8, m24, and m25, depending on sera (Figure 5E and 5F). For examples, serum No. 21 from an 11-year-old child with epilepsia was highly reactive with peptide m8. Most of the *Hp*-positive serum samples reacted with peptide m24 with variable reactivity. Among these samples, some reacted also with peptide m25 (Serum No. 3, No. 21 and No. 23) which overlapped m24 by five amino acids. Serum No. 20 from a child with juvenile rheumatoid arthritis, had strong non-specific reactions to GFP and IgG proteins.

The results of on-chip epitope mapping of the CagA-middle region with serum samples from 22 *Hp*-positive children are summarized in Table 3. Peptide m8 and peptide m24 reacted with 17 and 19 *Hp*-positive serum samples, respectively. The chip analysis indicated that there are at least two distinct epitopes in the middle region of CagA, represented by peptides m8 and m24–25.

**Table 3:** Epitope peptides in CagA^CPY2052^ middle region recognized by IgG antibodies in *H. pylori (Hp)*-positive child sera.

Peptides/proteins	Number of sera reacted in 22 *Hp*-positive sera (%)
**CagA-M peptides**	
m5	4
m8	17 (77%)
m13	1
m14	2
m15	1
m17*	0
m18*	7
m19	1
m20	1
m21	1
m24	19 (86%)
m25	5
**Proteins**	
GFP-CagA-M	22 (100%)
GFP	5

* Peptides reacted with one of Hp (-) serum samples.

**Figure 6:**
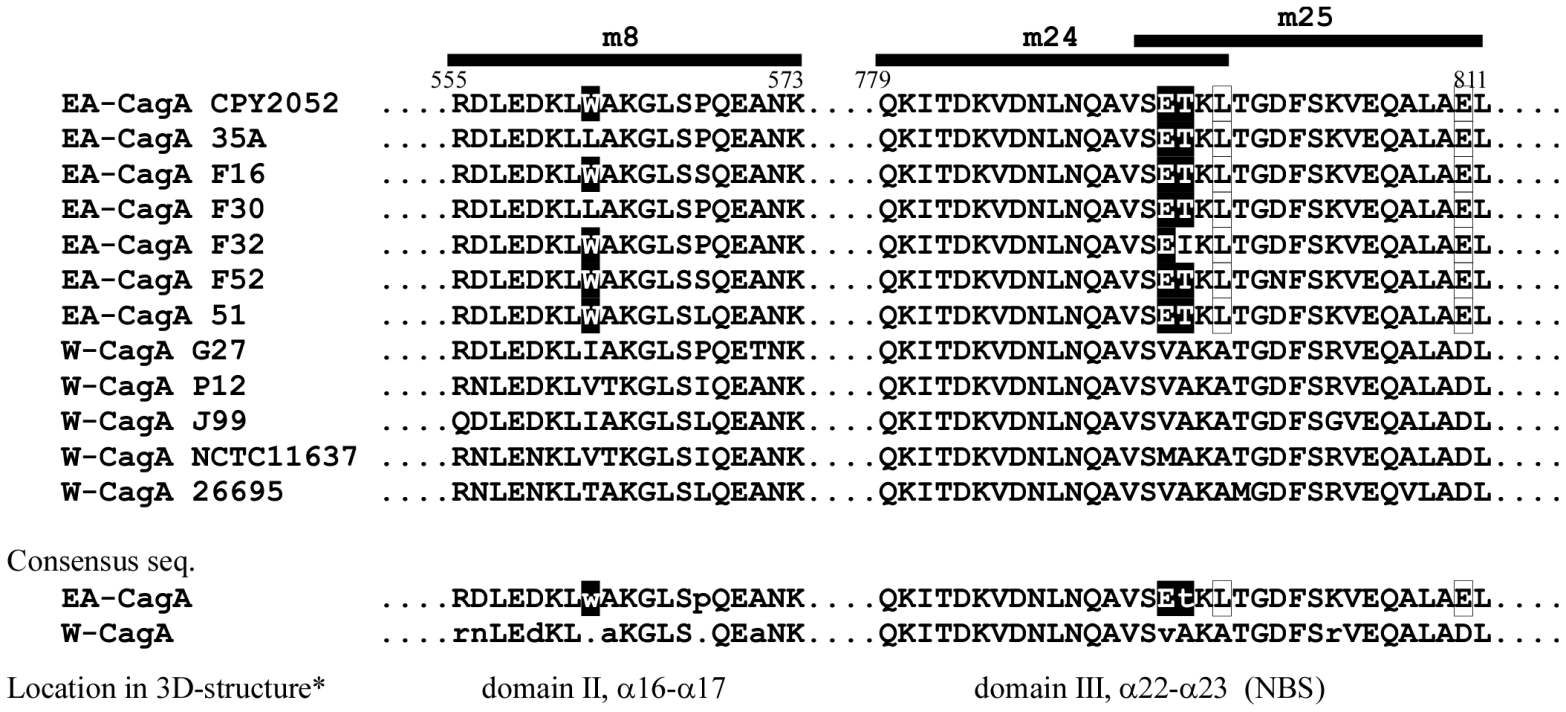
Alignment of epitope sequences in the CagA middle region and consensus sequences of epitopes in East Asian and western CagA. Amino acid sequences of seven East Asian and five western CagA corresponding to peptides m8 and m24–25 were taken from the NCBI database. Each sequence is denoted as EA-CagA (East Asian) or W-CagA (western) and written by single letters. Amino acids indicated with black boxes, tryptophan (W) in peptide m8 and glutamic acid (E) and- threonine (T) in peptide m24–25 are unique to EA-CagA. *: Location in tertiary structure of CagA^26695^ [36]. Consensus sequence among 7 EA-CagA or 5 W-CagA are shown by capital letters for all conserved amino acids, small letters for mainly conserved amino acids, or dot for no conserved amino acids.

The peptides m8 and m24-m25 analyzed were based on the sequence of CagA of *H. pylor*i strain CPY2052. CagA sequences of East Asian strains are known to differ from those of western strains [34], [35]. When the m8 and m24–25 peptides of published CagA sequences were aligned, remarkable differences were found (Fig. 6). The peptide m8 sequences contain a characteristic tryptophan residue in five out of seven East Asian CagA proteins, but none in western CagA proteins, though overall sequences are very similar. Similarly, the overlapping peptide m24–25 sequences of seven East Asian CagA contain a glutamic acid residue followed by threonine residue in six out of seven listed CagA that are remarkably different from non-polar valine, alanine and methionine residues in western CagA. Based on these results, we concluded that both fragment m8 and m24–25 contain epitopes specific to CagA of East Asian *H. pylori* strains.

## Discussion

Anti-*H. pylori* antibodies in sera from *Hp*-positive children were examined in this study. The 2-D immunoblotting and MS analysis identified 24 *H. pylori* candidate antigen proteins (Table 1). Some proteins had low amino acid coverage in this MS analysis, possibly because we were not able to analyze with the genome sequence of CPY2052 that is not available. We used the databases of NCBI and Swiss Prot, and manually incorporated protein databases of seven Mongoloid strains (five Japanese strains, a Korean strain, and an Amerindian strain).

The most dominant and ubiquitous antigen was CagA. It is noteworthy that sera of 14 *Hp*-positive asymptomatic children did not react with UreA, UreB, or GroEL, that are reported to be significant reactive antigens for antibodies in sera of adult patients [25]–[27], [29], [30]. We found that the serum reactivity of *Hp*-positive asymptomatic children depends on a few antigenic proteins.

The strong reactivity of CagA antibodies in sera from children were investigated via three methods: 1) 2-D immunoblot analysis of *H. pylori* lysate from wild-type and *cagA*- deletion strains, 2) 1-D immunoblot analysis of recombinant CagA fragments, and 3) peptide/protein-combined array analysis. CagA antibodies in sera from children were highly reactive with the middle region of CagA, and this reactivity was apparently depending on epitopes corresponding to peptides m8 and m24-m25 of CagA^CPY2052^. Both of the epitope regions contained sequences unique to East Asian CagA (Figure 6).

Recently, the 100 kDa-CagA fragment from western strain 26695 (1–876 from the total 1186 aa in the CagA^26695^) was stably crystallized and the precise structure was reported [36]. According to the report, m8 and m24–25 peptides are estimated to be located at the region of α-helix 16 to 17 in domain II, and α-helix 22 to 23 in domain III of CagA^26695^, respectively (Figure 6). In the tertiary structure, both the regions of α-helices are facing toward the outside of the CagA molecule. Additionally, the m24–25 peptides in α-helix 22 to 23 are located inside of the N-terminal binding sequence (NBS) of CagA^26695^ which is the special intermolecular interaction site that binds with the C-terminal binding sequence (CBS) located at the CagA^26695^ C-terminus [36]. Similarly with CagA^26695^ [36], [37], the C-terminal region of CagA^CPY2052^ appears to be very unstable as judged by the results of GFP-fused CagA fragments (Figure 4). Due to this instability, the m24–25 epitope in NBS would be exposed in the 100-kDa CagA fragment which is recognized by sera from Japanese *Hp*-positive individuals–both adult patients and asymptomatic children [17], [19]. The two epitopes, m8 and m24–25, in the middle region of the East Asian 100-kDa CagA fragment may be responsible for good performance of JHM-CAP kit that is derived from Japanese strains, specifically for serodiagnosis of young children. The potential role of epitope-specific CagA antibodies in *H. pylori*-infected children to develop *H. pylori*-related diseases must be studied in further investigations.

Finally, a new type of peptide/protein-combined chip was reported in this study. Recently, comprehensive linear peptide arrays were fabricated directly on membrane or on chips [38]–[40]. The 3-D structure of peptides can be expected to be kept by immobilization of pre-synthesized Cys-tagged peptides on flat maleimide-DLC chips. The N-terminal region of peptides are exposed in solution and directed away from the chip surface. This orientation would be the same as that of proteins arrayed on the maleimide-DLC chips [32], [33]. The newly devised maleimide-DLC peptide/protein chip technology described in this report may be applicable to epitope mapping of any antibody.

## Materials and Methods

### Ethics Statements

Written or verbal consents were obtained from the participants and/or their parent/guardian(s) for participation in this study. Verbal consent obtained for the early period serum collection in this study was recorded in the clinical card and each of the participants was asked to be involved in this study by mail, and confirmed their approval prior to the experiments. The public format explaining nature of this study, have been opened on the web site and as posters inside of Wakayama Rosai Hospital. The whole consent procedure was approved by the Institutional Review Boards (IRBs) of Yamaguchi University Hospital and of Wakayama-Rosai Hospital.

### Serum samples from children

Serum samples (n = 24) were collected between 1992 and 2002 at Wakayama-Rosai Hospital, Japan, and stored frozen until use. Stool antigen tests (Premier Platinum HpSA, Meridian Diagnostics Inc., Cincinnati, OH) and/or ^13^C-urea breath tests (UBT) were performed as described previously at Wakayama-Rosai Hospital to test for *H. pylori* infection. The ^13^C^–^Urea breath test (UBT) was performed same as described previously [41]. Of 24 children tested, 22 were *Hp-*positive and two were *Hp-*negative. *Hp-*positive children ranged from 2–13 years old (average age 7.9), 14 were asymptomatic, and eight were symptomatic (two with juvenile rheumatoid arthritis (Serum No. 15, and 20), one with iron-deficiency anemia (No. 22), three with epilepsy (No. 4, 21, and 23), one with epilepsy and iron-deficiency anemia (No. 11), and one with atrophic gastritis, epilepsy and anemia (No. 19)). *Hp-*negative children were ages 7 and 9 years old, one was apparently asymptomatic (No. 17) and the other had pylorostenosis (No. 24). The experimental protocol, which involved clinical serum samples, was reviewed and approved by the Institutional Review Board of Yamaguchi University Hospital and Wakayama-Rosai Hospital.

### *H. Pylori* strains and growth conditions

Japanese *H. pylori* strains, CPY2052 and HPK5, both isolated from gastric ulcer patients, and CPY3401 isolated from a duodenal ulcer patient at Yamaguchi University Hospital, Japan [42], and western reference strains, NCTC11637 and 26695, were used in this study. The *cagA* DNA sequence and deduced amino acid sequence of CPY2052 was deposited at NCBI GeneBank (Accession numbers DQ091000 and AAZ23952, respectively). Isogenic *cagA* deletion strains and *vacA* deletion strains from CPY2052 and NCTC11637 were constructed as described previously [43]. Isogenic *cagA* deletion strains of HPK5 were constructed in this study by the same procedure. Strains of *H. pylori* were cultured for 2 days under 10% CO_2_ at 37°C on Brucella agar plates supplemented with 5% fetal bovine serum (FBS), 10 µg/mL vancomycin, and 2 µg/mL amphotericin B. The protocol of *H. pylori* genetic engineering experiments were reviewed and approved by the Institutional Review Board of Yamaguchi University.

### *H. Pylori* lysate preparation, SDS-PAGE and two-dimensional gel electrophoresis (2-DE)

*H. pylori* bacteria from one 10-cm plate culture were suspended in cold 10% glycerol, washed once, pelleted and freeze-stocked at −80°C until use. For SDS-PAGE, the bacterial pellet was dissolved well by pipetting in SDS sample buffer. Each lysate containing 10 µg proteins was loaded onto a 2–15% gradient polyacrylamide gel (Cosmo-Bio, Tokyo, Japan).

The bacterial pellet was dissolved well by pipetting in 2-DE lysis buffer (8 M urea, 4% CHAPS, 40 mM Trisma base, 10 µg/mL aprotinin, 10 µg/mL leupeptin, and 1 mM PMSF); each mixture was left at room temperature for 30 min, and then centrifuged at 21,500×g for 15 min at room temperature; each resulting supernatant was obtained as the lysate samples for 2-DE, although a pellet was rarely observed. Protein concentrations of each lysate solution were measured with RC-DC Protein Assay kits (Bio-Rad, Hercules, CA) according to the manufacturer’s instructions. The samples were stored at −80°C until use.

The first-dimensional isoelectric focusing (IEF) was carried out on 7 cm pH 3–10 immobilized linear gradient (IPG) strips (Bio-Rad). Strips were incubated overnight in a re-swelling tray (GE healthcare Japan, Tokyo, Japan) with re-hydration solution (8 M urea, 2% CHAPS, 2 M thiourea, 2.3% triton X-100, 0.0023% ASB-14, 1.4% De-streaking solution (GE), and 0.5% IPG buffer (GE)). On each re-hydrated IPG strip, 300 µg (CPY2052 and its isogenic mutant) or 50 µg (other strains) of *H. pylori* lysate proteins were loaded in a cup (by cup loading method) at the anode side. IEF was run using IPGphor3 (GE) in five steps: 500 V gradient for 30 min, 1000 V gradient for 1 h, 4000 V gradient for 1.5 h, and 5000 V step in hold for 45 min, 500 V step in hold for several h until 14000–18000 Vh total. Following IEF, two equilibrium steps of 2-ME and iodoacetamide were carried out following the manufacture’s instructions (GE). The second-dimension was performed on 8–16% gradient polyacrylamide gel (Cosmo-Bio), in SDS-running buffer with 15 mA/gel to separate constituent proteins. SeePico CBB staining Kit (Benebiosis, Seoul, Korea) was used to stain the gels.

### Immunoblot analysis

The SDS-PAGE or the 2-DE gels were blotted onto PVDF membranes (Millipore, Billerica, MA, USA) with a wet-transfer system (Bio-Craft, Tokyo, Japan) and tank-transfer buffer (25 mM Tris-HCl, 192 mM glycine, 15% methanol, and 0.005% SDS) at 100 V constant for 2 h followed by cooling in ice-water. The membrane was blocked for 3 h with the blocking solution containing 0.05% Tween-20 in TBS (TBS-T) supplemented with 5% bovine serum albumin and 0.02% sodium azide, and incubated with 1∶1,000 diluted serum from each child in blocking solution for 16 h at 4°C. Control antibodies used were, rabbit anti-East Asia CagA specific antibody (anti-EAS) [44], a rabbit anti-CagA antibody (Austral, San Roman, CA) raised against C-terminal Glu748 to Glu1015 of western CagA (anti-western-CagA-C), and rabbit anti-VacA antibody [45]. Incubations for immunoblotting were followed by washes and subsequent incubations with 1∶5000 diluted horseradish peroxidase-conjugated goat anti-rabbit IgG (Jackson ImmunoResearch Laboratories, West Grove, PA). After 10 sequential washes with TBS-T, each membrane was incubated with 1∶2000 diluted horseradish peroxidase-conjugated goat anti-human IgG (Jackson), incubated with blocking solution for 2 h, and washed five times with TBS-T. The reaction was visualized with the chemiluminescence reagent ImmunoStar HD (Wako, Osaka, Japan) under a luminescence imager LAS-1000 (Fuji Film, Tokyo, Japan). After the luminodetection, each membrane was washed with distilled water, dipped in methanol, and stained in a solution of 2% CBB, 10% acetic acid, and 50% methanol for 2 min to take CBB-stained image. The membrane was de-stained with 10% acetic acid and 50% methanol solution. The Progenesis Samespots software (Nonlinear Dynamics, Newcastle, UK) and the imaging software Canvas X (ACD system of America, Miami, FL) were used to superimpose a CBB-stained membrane image with the immunodetection image of the same membrane. The superimposed images above and SeePico CBB stained gels were also matched following the procedure described above. Distinguished antigenic spots were cut from the gels and subjected to LC-MS/MS analysis.

### In-gel protein digestion and LC-MS/MS analysis

In-gel digestion by trypsin and the following LC-MS/MS analysis using the Agilent 1100 series LC/MSD Trap XCT (Agilent Technologies, Palo Alto, CA) were performed as described previously [46]. Proteins were identified by the Spectrum Mill MS Proteomics Workbench against the Swiss-Prot protein database search engine, and the NCBI protein database search engine. We also manually incorporated protein information from seven *H. pylori*-specific genome databases (five Japanese strains 35A, F16, F30, F32, F52, a Korean strain Hp51, and an Amerindian strain Shi470) that were downloaded from NCBI Genome site of *Helicobacter pylori*.

### *E. coli* lysate containing partial CagA recombinant protein

Plasmid were constructed in *E. coli* DH5α cells to generate eight GFP-fused CagA fragments, each containing a distinctive CagA fragment, N1, N2, ‘NM, M, MC’, C3, C2, or C1, a 6x histidine (6xHis)tag at the N-terminus, and a 5x cysteine (5xCys)-tag at the C-terminus (Figure 4A). Recombinant proteins were expressed in *E. coli* BL21 at 28°C for 18 h as described previously [33]. Whole-cell lysate prepared from aliquots of bacterial pellet was 0.1 to 10 µL out of 200 µL total lysates for 1 mL culture, was adjusted depending on the corresponding recombinant protein band in the SDS-PAGE of See Pico CBB-stained gel (Fig. 2B). The immunoblot analysis of the *E. coli* lysates by child sera was performed as described in the *immunoblot Analysis* section.

### Peptide/protein chip preparation

Each of proteins, 6xHis-GFP-CagA-M-5xCys, 6xHis-GFP-5xCys, 6xHis-GFP-human HSP70-N-teraminus-5xCys, and 6xHis-GFP-human HSP70-C-teraminus-5xCys, were expressed in *E. coli* strain BL21 and purified using the 6xHis-tag as described previously [32], [33]. The protein concentration was adjusted to 25.6 µmol/mL in PBS. A series of 25 peptides (m1-m25 in Fig. 4A) were designed and synthesized (>70% purity, 5 mg) commercially (Scrum, Tokyo, Japan), each constituted 22 amino acids (19 from the CagA-M sequence and 3xCys at the C-terminus) and overlapped by five amino acids of the adjacent peptides. Peptides were dissolved in PBS and adjusted to 2 pmol/µL solution. Human IgG, rabbit IgG and mouse IgG were dissolved and adjusted to 12.8 µmol/mL in PBS. Each peptide/protein solution was prepared for immobilization as described previously [33], and was printed on 3×3 mm maleimide-incorporated diamond-like carbon (DLC)-coated silicon chip substrate (Toyo Kohan, Tokyo, Japan), as 10 nL spots (approximately 200 µm diameter), containing 128 fmol each for recombinant proteins, 10 fmol each for peptides, and 64 fmol each for control IgGs.

### Epitope mapping of serum antibodies against CagA-middle region, and peptide alignments

Antibody detection was performed as described previously [32] with some modifications. Briefly, chips were dipped in 100 µL of SDS/2ME sample buffer, heated at 95°C for 5 min, washed five times in TBS-T, and incubated in blocking solution (1.5 h), washed, incubated in 1∶30 diluted serum in 100 µl blocking solution (1.5 h), washed, and in 1∶500 diluted Alexa Fluor 633 (Alexa633)-conjugated anti-human IgG antibody (Invitrogen) (1 h). For control spots, anti-GFP (1∶1000, Santa Cruz Biotechnology), anti-FLAG (F2, 1∶3000, Sigma-Aldrich, St. Louis, MO) and 1∶500 diluted Alexa Fluor 555 (Alexa555)-conjugated anti-mouse IgG antibody (Invitrogen, Carlsbad, CA) was used. Chips were washed and placed on a chip holder, and mounted with PBS and a cover slip for image analysis.

Fluorescent images were obtained by a microarray scanner GenePix 4000B (Molecular Devices, Sunnyvale, CA). Two control human IgG spots were used to monitor secondary antibody detection, and chips detection in full-color mode were adjusted yellow in each chip to compare each positive spots among other chips.

CagA sequences of seven East Asian *H. pylori* strains and five western *H. pylori* strains were downloaded from NCBI genome sites. ClustalW Alignment and Accelrys Gene ver. 2.5 (Accelrys, San Diego, CA) were used to align 12 CagA amino acid sequences.

## Supporting Information

Table S1 **Grouping of** ***H. pylori*** **antigenic proteins recognized by IgG antibodies in sera of Japanese children on the base of age and disease context.**
(DOCX)
