# Supplementary material for: Proteomic Characterization of Helicobacter pylori CagA Antigen Recognized by Child Serum Antibodies and Its Epitope Mapping by Peptide Array
Source: PLoS One. 2014 Aug 20;9(8):e104611. doi: 10.1371/journal.pone.0104611 (PMC4139317; doi:10.1371/journal.pone.0104611)
Supplement: Supplementary file 1 [file pone.0104611.s001.docx]

	W26	FlaA	●	●	-	●	●	●	●	●	-	-	●	-	●	●	●	●	●	●	●	●	-	●	●
	W53		-	-	●	●	●	●	●	●	-	●	-	●	●	●	●	●	●	●	●	-	●	-	-
	W2	VacA	-	-	-	-	-	-	●	●	●	-	-	-	-	●	●	●	-	●	●	-	●	-	●
II	W12		-	-	-	●	-	●	-	●	-	-	●	-	-	-	-	●	-	-	-	-	-	-	-
	W57	HpaA, UreA	-	-	-	-	-	-	-	●	-	-	●	-	●	●	-	●	●	-	●	-	-	-	-
	W45		-	-	●	-	●	-	-	-	-	●	-	●	-	-	-	-	-	-	-	-	-	-	●
	W52	RfaD	-	-	-	-	●	-	●	●	-	-	-	-	-	-	-	-	●	-	-	-	-	-	-
	W38	Tpx<br>Ggt fragment	-	-	-	●	-	-	-	-	-	-	●	-	-	-	-	-	●	-	-	-	-	-	-
	W69	MCP, HslU	-	-	-	-	-	-	-	-	●	-	●	-	-	-	-	-	-	-	●	-	-	●	-
	W18	ScoA	-	-	-	-	-	-	-	-	-	-	●	-	-	-	-	-	●	-	-	-	-	-	-
	W50		-	-	-	-	-	-	-	-	-	-	-	-	-	-	-	●	-	-	-	-	-	-	●
	W76		-	-	-	-	-	-	-	-	-	-	-	-	-	●	-	-	-	-	-	-	-	-	-
III	W60	Omp21/AlpB	-	-	-	●	-	●	-	-	-	-	-	●	-	●	-	-	●	●	●	●	●	●	●
	W9	HefB	-	-	-	-	-	-	●	-	-	-	-	-	-	-	●	-	-	●	-	●	●	●	●
	W25	FusA	-	-	-	-	-	-	-	-	-	-	-	-	-	●	-	-	-	●	●	-	-	●	-
	W51	Omp18	-	-	-	●	-	-	-	-	-	-	-	-	-	-	-	-	●	●	-	-	-	-	-
IV	W4	PorB	-	-	-	-	-	-	-	-	-	-	-	-	-	-	-	-	●	●	-	●	-	●	●
	W33	UreB	-	-	-	-	-	-	-	-	-	-	-	-	-	-	-	-	●	●	●	-	-	●	●
	W37,58,59	UreA	-	-	-	-	-	-	-	-	-	-	-	-	-	-	-	-	●	●	-	●	-	●	●
	W34	GroEL	-	-	-	-	-	-	-	-	-	-	-	-	-	-	-	-	-	●	●	●	-	●	-
	W77	Omp27/HopQ	-	-	-	-	-	-	-	-	-	-	-	-	-	-	-	-	-	●	-	-	●	●	-
	W3	HyuA	-	-	-	-	-	-	-	-	-	-	-	-	-	-	-	-	●	-	-	-	-	-	●
	W85		-	-	-	-	-	-	-	-	-	-	-	-	-	-	-	-	-	-	●	-	-	-	●
	W84	GuaB	-	-	-	-	-	-	-	-	-	-	-	-	-	-	-	-	-	-	-	-	-	●	●
Total number of serum-reacted spots listed			1	1	4	8	5	6	7	9	4	4	8	5	5	9	5	8	14	14	11	8	7	12	13
Disease*/ Sum of reacted spots listed				P															R	R	E	E	IE	E	I

●: reacted, -: not reacted.

*: R: juvenile rheumatoid arthritis, E: epilepsy, I: iron-deficiency anemia, G: atrophic gastritis, A: anemia, P: pylorostenosis
